# Efficacy of multi-domain cognitive function training on cognitive function, working memory, attention, and coordination in older adults with mild cognitive impairment and mild dementia: A one-year prospective randomised controlled trial

**DOI:** 10.7189/jogh.13.04069

**Published:** 2023-06-30

**Authors:** Chien-Mei Sung, Tso-Ying Lee, Hsin Chu, Doresses Liu, Hui-Chen Lin, Li-Chung Pien, Hsiu-Ju Jen, Yueh-Jung Lai, Xiao Linda Kang, Kuei-Ru Chou

**Affiliations:** 1 School of Nursing, College of Nursing, Taipei Medical University, Taipei, Taiwan; 2 Department of Nursing, Fu Jen Catholic University Hospital, Fu Jen Catholic University, New Taipei City, Taiwan; 3 Nursing Research Center, Taipei Medical University Hospital, Taipei, Taiwan; 4 Institute of Aerospace and Undersea Medicine, School of Medicine, National Defense Medical Center, Taipei, Taiwan; 5 Department of Neurology, Tri-Service General Hospital, National Defense Medical Center, Taipei, Taiwan; 6 Department of Nursing, Wan Fang Hospital, Taipei Medical University, Taipei, Taiwan; 7 Research Center in Nursing Clinical Practice, Wan Fang Hospital, Taipei Medical University, Taipei, Taiwan; 8 Post-Baccalaureate Program in Nursing, College of Nursing, Taipei Medical University, Taipei, Taiwan; 9 Psychiatric Research Center, Wan Fang Hospital, Taipei Medical University, Taipei, Taiwan; 10 Department of Nursing, Taipei Medical University-Shuang Ho Hospital, New Taipei, Taiwan; 11 School of Nursing, University of Pennsylvania, Philadelphia, USA; 12 Psychiatric Research Center, Taipei Medical University Hospital, Taipei, Taiwan; 13 Neuroscience Research Center, Taipei Medical University, Taipei, Taiwan

## Abstract

**Background:**

Cognitive function, working memory, attention, and coordination are higher-level functions sharing a complex relationship. Limited evidence exists on the effectiveness of multi-domain cognitive function interventions to improve cognitive outcomes. We evaluated the effectiveness of such interventions on cognitive function, working memory, attention, and coordination in older adults with mild cognitive impairment and mild dementia.

**Methods:**

We conducted a double-blind, two-arm, parallel-group randomised controlled trial in community care centres of Northern Taiwan. We recruited 72 participants aged*≥*65 years and randomly allocated them using 1:1 block randomization (block size = 4) into experimental (multi-domain cognitive function training) (MCFT) and control groups (passive information activities) (PIA) (n = 36/group). We administered the interventions in both groups for 30 minutes per session, three sessions per week for eight weeks, for a total of 24 sessions. The outcome indicators were cognitive function assessed (mini-mental status examination), working memory (digit span), selective attention (Stroop test), visual-spatial attention (trail making test-A (TMT-A)), divided attention (trail making test-B (TMT-B)), and coordination (Berry visual-motor integration (Berry-VMI)). We evaluated the study outcomes at baseline, immediate post-test, one-month follow-up, and one-year follow-up.

**Results:**

We found no significant differences between the groups at baseline except for education. The average age of participants was 82.3 years, and most (76.4%) were female. We analysed the results by generalised estimating equations (GEE) based on the intention-to-treat (ITT) principle. The multi-domain cognitive function training was effective in improving cognitive function (β = 1.7; 95% confidence interval (CI) = 0.63-2.31; *P* = 0.001), working memory (β = -1.45; 95% CI = -2.62, -0.27; *P* = 0.016), and selective attention (β = -23.3; 95% = CI -43.9, -2.76; *P* = 0.026) compared to passive information activities at 1-month follow-up. The effects of multi-domain cognitive function training on cognitive function (β = 1.51; 95% CI = 0.40-2.63; *P* = 0.008), working memory (β = -1.93; 95% CI -3.33, -0.54; *P* = 0.007), selective attention (β = -27.8; 95% CI = -47.1, -8.48; *P* = 0.005), and coordination (β = 1.61; 95% CI = 0.25, 2.96; *P* = 0.020) were maintained for one year. There were no significant improvements in attention outcomes (visual-spatial and divided attention) after training.

**Conclusions:**

MCFT intervention demonstrated favourable effects in improving global cognitive function, working memory, selective attention, and coordination among older adults with mild cognitive impairment and mild dementia. Thus, applying multi-domain cognitive training in older adults with mild cognitive impairment and mild dementia could help to delay the cognitive decline.

**Registration:**

Chinese Clinical Trial Registry (ChiCTR2000039306).

Rapid global population aging has led to a considerable increase in the number of people with dementia, which was estimated at 50 million by the World Alzheimer Report 2021 with an expected increase to 152 million by 2050. As diseases progress in older adults, significant resources are invested for treatment, with an estimated cost of care at US$1 trillion annually, which is expected to double by 2030 [1]. Thus, dementia care also generates a financial burden for society.

Mild cognitive impairment (MCI) is a transitional period before the decline in cognitive function from normal ageing progresses to dementia. Approximately 10%-15% of people with a clinical diagnosis of MCI develop dementia each year [2]. MCI and mild dementia are characterised by objective evidence of cognitive impairment. Working memory, attention, and executive function of the brain are the first to deteriorate in patients with MCI, with the first being the most prominent manifestation of poor cognitive function [3]. Working memory and executive function are higher-order cognitive functions used to perform various tasks such as calculation, planning, inference, reasoning, comprehension, learning, and attention control. There are also many similarities in diagnosis and recognition between MCI and mild dementia, with mild dementia involving more than one cognitive domain and substantially interfering with daily life. Regarding socio-economic costs, it is important to maintain the independent activities of daily living of patients with MCI or mild dementia to reduce financial burden in communities and families. Previous studies have indicated that to promote brain plasticity, interventions including cognitive training that activate the brain’s frontal lobe and improve attention and memory must be implemented to maintain and improve target function [4,5]. Additionally, brain stimulation through cognitive activities helps establish new connections between neurons and increases neuroplasticity, thereby enhancing cognitive reserve and maintaining or improving its cognitive function [6].

Evidence shows that active cognitive training may delay cognitive aging. Findings from randomised controlled trials [7-11] and meta-analyses [12-14] have demonstrated that positive effects of cognitive training mostly cover a single domain and improve cognitive performance (such as working memory, attention, executive function, and coordination) after six months, but more research is needed for multi-domain cognitive training’s effects on cognitive functioning. In the Advanced Cognitive Training for Independent and Vital Elderly (ACTIVE) study, cognitive training was shown to improve cognitive performance in community-dwelling older adults immediately after training and after follow-ups of two, five, and even 10 years [15]. Training resulted in cognitive improvement only within the targeted cognitive domain. However, cognitive functions are not independent, with some overlapping and others requiring coordination [16]. For example, attention, memory, and executive function are functionally related and exhibit similar characteristic of sharing neural circuits that overlap and interact in a complex relationship, which cannot be performed independently [17]. There are only a few studies on multi-domain cognitive function interventions, which mostly include healthy older adults, and there is limited research focusing on individuals with mild cognitive impairment. According to the Scaffolding Theory of Aging and Cognition (STAC), based on the principles of brain plasticity, aging can lead to changes in an individual's neurological and cognitive functioning [18]. However, engaging in activities such as learning tasks, exercise, and cognitive training can enhance their neurological functioning and subsequently impact their level of cognition. There are various methods of cognitive training intervention, including stimulation, rehabilitation, and cognitive training itself; while they differ in approaches and objectives, they all aim to promote cognitive functioning. The key distinction of multi-domain cognitive training from other cognitive interventions lies in its ability to account for the intricate interplay between multiple mental processes necessary to maintain an adaptable mental state that allows individuals to interact appropriately with their environment.

Building on the success of ACTIVE, multi-domain cognitive function training (MCFT) has been developed and tested. We used a brain plasticity-based adaptive cognitive training developed by LTPA (leisure time physical activity). This game-based training was designed to stimulate the cerebral cortex to improve cognitive functioning of patients. As such, older adults with MCI and mild dementia are a crucial population to intervene as evidence has shown that cognitive training can delay rapid cognitive function decline. Therefore, we conducted the first randomised controlled trial to explore and evaluate the efficacy of a short-term MCFT compared to passive information activities (PIA) training in older adults with MCI and mild dementia. We examined the cognitive function, working memory, attention, and coordination among older adults with MCI and mild dementia in the primary analyses. To examine the long-term effects of the MCFT and PIA on cognitive function, working memory, attention, and coordination, we evaluated the patients throughout a one year period.

## METHODS

### Study design and setting

We conducted a double-blind, two-armed, parallel group randomised controlled trial with repeated measures to examine the effects of MCFT for older adults with MCI and mild dementia. We registered the study with the Chinese Clinical Trial Registry (ChiCTR2000039306, 5 November 2020), recruiting the first participant on 16 November 2020.

We recruited older adults aged ≥65 years with MCI and mild dementia from nine community care centres in Northern Taiwan between November 2020 and March 2022, where trained interviewers screened the participants for eligibility. Then, the participants were informed of the study’s aim and methods and asked to sign a consent form. We collected the participants’ demographic and sensory function (visual and auditory) data through structured questionnaires. We included older adult ≥65 years, provided they had a Clinical Dementia Rating (CDR) of 0.5 or 1 point, were able to move independently and had no physical disabilities, were able to communicate, and wished to participate voluntarily after providing informed consent. We excluded individuals with a history of other cognitive, memory, attention, or coordination training in the previous year, history of head trauma, loss of consciousness, stroke, or rapid cognitive decline, diagnosis of severe mental illness or behavioural problem resulting in the inability to undergo training, and severe visual, hearing, or communication impairment.

### Randomisation and masking

We coded and grouped the eligible participant prior to randomisation. A research assistant not involved in the study generated the randomisation sequence with an online randomization program, after which block randomization (block size = 4) was adopted to ensure each group contained an equal number of participants, preventing the participants or the researchers from identifying the order of group allocation (ratio = 1:1). The research assistant placed a labelled card indicating the random group allocation (1 – experimental group, 2 – control group) into sequentially numbered sealed opaque envelopes. We adopted a a double-blinded design, informing the participants that two interventions would be implemented but that they would not know to which one they were assigned. We did not disclose the allocation process and other relevant information to the research personnel. To maintain blinding of measurement, outcome evaluators could not discuss the content or participant allocation with any relevant personnel and were only responsible for data collection and analysis of the results.

### Intervention group

The game-based intelligence test (developed by LTPA solution Co., LTD) with an “audio-visual interactive input interface device” and a “special training course application (APP) on a smart phone or tablet” provides a multi-domain cognitive intelligence system that was used to train experimental group’s memory, attention, and coordination. A research assistant first created an account with their progress being logged in the system database and visualised through charts, after which he selected the training category in the APP and operated the sound and light feedback module. The light feedback function was used to track the long-term effects of the training. The difficulty level of the game was adjusted from one to 10 depending on each participant’s abilities (e.g. reaction time). Based on previous study findings, the training programs in both groups were set to be performed two to three times a week for six to eight weeks [7,11,12,19-21]. The MCFT group received 30-minute individual-format training sessions three times a week for eight weeks with a total of 24 sessions. We trained the participants in both groups at different times and they could not contact each other.

#### Memory training

One to four balls would randomly light up for one to three seconds as a reminder and then turn off, and a sound would prompt the participants to tap the balls in the order in which they lit up. We recorded the participants’ performance and number of taps. This training was administered to improve the participants’ visual working memory and ability to quickly store and process information.

#### Attention training

Two balls would light up simultaneously in different colours. The participants were required to identify one colour designated as the target (selective attention). When the participants tapped the correct ball, both lights would disappear; we recorded all errors. This training was administered to improve the participants’ visual search abilities (visual-spatial attention) by helping them focus on a task for a certain period (sustained attention), increasing their attention span, and decreasing reaction time, which can strengthen problem-solving abilities.

#### Coordination training

The participants would tap the illuminating balls as they lit up; we recorded their reaction times. This training was administered for the participants to use their eyes to track dynamic objects to improve their eye-hand coordination and coordination, which strengthens the ability to pick up and manipulate everyday objects and control movement.

#### Control group

The control group underwent 24 sessions of individual-format PIA comprised of listening to audio books on the Himalaya APP and reading newspapers. We conducted pre-test assessments in both groups; the scores served as the benchmark for the data analysis. We collected post-test performance indicators immediately after the eight-week intervention, one-month follow-up, and one-year follow-up.

### Data collection

We collected demographic characteristics at pre-test using a structured form, which included age, sex, marital status, education, living situation, chronic disease, and dementia (family history). We collected data on other outcome measures at four time points: pretest (T0), eight-week post-test (T1), one-month follow-up (T2) and one-year follow-up (T3).

### Outcome measures

We used more than one primary endpoint of several outcome measures of equal therapeutic importance. The primary outcome indicators were global cognitive function, working memory, attention, and coordination. The secondary outcome indicator was sleep quality. Due to length limitations for publications, here we only present the primary outcome indicators.

#### Global cognitive function

The mini-mental status examination (MMSE) [22] is a quantitative scale used to evaluate cognitive ability concerning orientation, attention, calculation, recall, visual-spatial ability, and language. The MMSE consists of 11 items, and the total possible score is 30 points. Higher scores indicate stronger abilities. Studies have noted that the MMSE is easy to use and has high reliability and validity. The sensitivity is 0.88 and 0.84, the ratio is 0.70 and 0.86, the area under the curve to distinguish mild cognitive impairment from mild dementia is 0.88 and 0.89, the positive predictive values are 0.94 and 0.80, the negative predictive values are 0.81 and 0.88, Cronbach’s alpha is 0.68 to 0.96, the test-retest reliability is 0.74 to 0.99, and the interrater reliability is 0.83 [22-24].

#### Working memory

We measured the working memory through the digit span test (DST) with forward digit span (DS-forward) and backward digit span (DS-backward). A string of numbers starting with two digits and then gradually increasing in length are read to the participants. The test was performed twice. The participants listened carefully to one string of random numbers per second. The DS-forward assessment required the participants to repeat the numerical strings in the exact order, and the DS-backward required the participants to repeat the number strings in reverse order. These types of tests are commonly used to assess defects in short-term memory capacity. DST forward and backwards tests can quickly and easily measure performance in short-term memory capacity, providing a basis for diagnosis, and are a widely accepted, research-proven method to measure mild cognitive impairment. The test had no time limit and exhibited favourable internal consistency (0.85-0.99) and test-retest reliability (0.75-0.99) [25].

#### Selective attention

The Stroop colour and word test (SCWT) is a neuropsychological test used for experimental and clinical purposes to assess cognitive interference suppression, which occurs when the processing of one aspect of a stimulus affects the processing of another aspect of the stimulus [26]. In the initial version of SCWT proposed by Stroop in 1935, participants were required to read three tables as quickly as possible. SCWT consisted of three sections, namely words, colours, and colour words. In each section, participants were presented with a page consisting of 10 columns, each containing 10 items, for a total of 100 items per page. The score for each section was the total number of words read within 45 seconds. This test exhibits high reliability (*r*>0.80) [27].

#### Divided and visual-spatial attention

We used the trail making test (TMT) to assess divided and visual-spatial attention. The TMT comprises parts A and B consisting of 25 circles each on a sheet of A4 paper. In part A, the circles are numbered one to 25 (in each circle), and the participants drew lines to connect them in numerical order. In part B, the circles contain numbers from one to 13 and 12 Chinese zodiac signs (e.g. rat, ox, and tiger). As in part A, the participants drew lines connecting the circles in ascending numerical order but alternated between numbers and zodiac signs (i.e. one-rat-two-ox-three-tiger) [28-30]. The score for each section was the time required to complete the task in seconds. The TMT-A was used to measure visual-spatial attention, while TMT-B was used to measure divided attention. The inter-rater reliabilities of part A and B were 0.996 and 0.998, respectively [31].

#### Coordination

The Beery-Buktenica developmental test of visual-motor integration (Beery-VMI) consists of three parts: visual-motor integration, visual perception, and motor coordination. It requires integration and emphasises the sequential nature of the participants’ capability development. Additionally, it unaffected by cultural context. We only evaluated the participants’ motor coordination. The Beery-VMI consists of geometric designs that increase in complexity. Higher scores indicate higher visual-motor integration, and the test has no time limit [32]. The one-week test-retest reliabilities of the Beery-VMI, visual perception, and motor coordination portions were 0.88, 0.86, and 0.84, respectively, for those aged 20 and 60-69 years. The overall reliabilities of the Beery-VMI, visual perception, and motor coordination portions were 0.88, 0.88, and 0.90, respectively [33].

### Statistical analysis

We used SPSS (version 26.0, SPSS Inc., Chicago, IL, USA) for data filing and statistical analysis. We used independent *t*-tests and χ^2^ tests to compare the descriptive data from the control and intervention group at baseline (T0). We repeatedly measured intervention effectiveness using generalised estimating equation (GEE) analysis to identify time-dependent changes in outcome variables by examining the efficacy of MCFT and comparing the differences between the two groups. We considered *P*-value of <0.05 statistically significant. We monitored therapeutic adherence, sample attrition rate, medication compliance, and adverse events during the study period. We managed missing data according to the intention-to treat (ITT) principle, ensuring that the outcome data analysis accounts for the initial random assignment into the intervention and control groups.

### Ethical considerations

The respective review boards at the Taipei Medical University Joint Institutional (N201909018) approved the study. We provided all participants with an information sheet about the study prior to enrolment and obtained signed consent forms. Participation was fully voluntary. We assured the participants about safety issues, informing them of their right to withdraw or discontinue participation at any time without penalty, and that the collected data would remain strictly confidential and would be used only for research purpose. No personal information would appear in the research reports.

### Sample size estimation

We estimated the sample size using the G*Power statistics software (Version 3.1.9.4) and adopting a priori power calculations (repeated measures, between factors, to obtain an α of 0.05, a power of 0.8, and effect size *f* of 0.3 with two groups and four measurements) based on prior research [7]. We needed 58 participants to detect significance. However, with the study’s one-year follow-up and the impact of the COVID-19 pandemic, we set the sample size to 72 participants, with 36 participants per group after accounting for a 25% attrition rate.

## RESULTS

**Figure 1:**
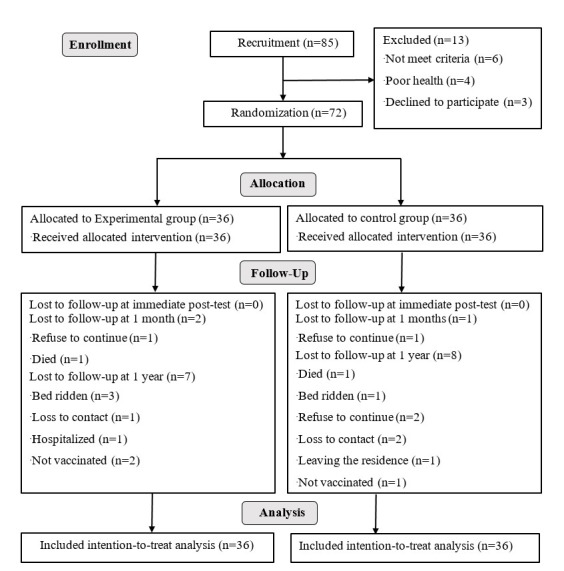
Participant enrolment according to the CONSORT 2010 flow diagram.

We initially enrolled 85 participants; seven refused to participate after being informed of the study aim and six had dementia and were thus ineligible. Finally, we included 72 eligible participants and randomly allocated them to either the MFCT group or control group (n = 36/group) (**Figure 1**).

### Participant demographics

Fifty-five participants were women and 17 were men, with a mean age of 82.3 years (standard deviation (SD) = 6.46) and no difference observed between the groups (*P* = 0.953). The average MMSE scores was 21 (SD = 3.18), indicating that most participants did not have a major cognitive impairment. Most of the subjects in both groups were classified with MCI (91.7%) with no significant difference between the two groups (*P* = 0.498). The groups did not differ significantly in any other demographic variables except for education, with a borderline significance for sex (**Table 1**). We adjusted the GEE models for education.

**Table 1:** Demographic characteristic and between-groups comparisons of the participants in baseline (T0)*

Variable	Total (n = 72)	MCFT group (n = 36)	Control group (n = 36)	*P-*value
**Age in years, mean (SD)†**	82.3 (6.46)	83.5 (6.36)	81.0 (6.41)	0.953
MMSE, mean (SD)**†**	21 (3.18)	20.5 (2.93)	21.7 (3.32)	0.498
MCI	66 (91.7)	33 (91.7)	33 (91.7)	
Mild dementia	6 (8.3)	3 (8.3)	3 (8.3)	
**Sex**				0.053
Female	55 (76.4)	31 (86.1)	24 (66.7)	
Male	17 (23.6)	5 (13.9)	12(33.3)	
**Marital status†**				0.099
Married	35 (48.6)	14 (38.9)	21 (58.3)	
Widowed	37 (51.4)	22 (61.1)	15 (41.7)	
**Education‡**				0.001
Illiterate	30 (41.7)	23 (63.9)	7 (19.4)	
Primary school	23 (31.9)	10 (27.8)	13 (36.1)	
Secondary school or above	19 (26.4)	3 (8.3)	16 (44.4)	

MCFT – multi-domain cognitive function training, MMSE – mini-mental state examination, MCI – mild cognitive impairment

*Data are presented as frequency (%) unless otherwise specified.

**†**Independent *t*-test.

‡χ^2^ test.

### Effects of intervention on global cognitive function (MMSE)

**Figure 2:**
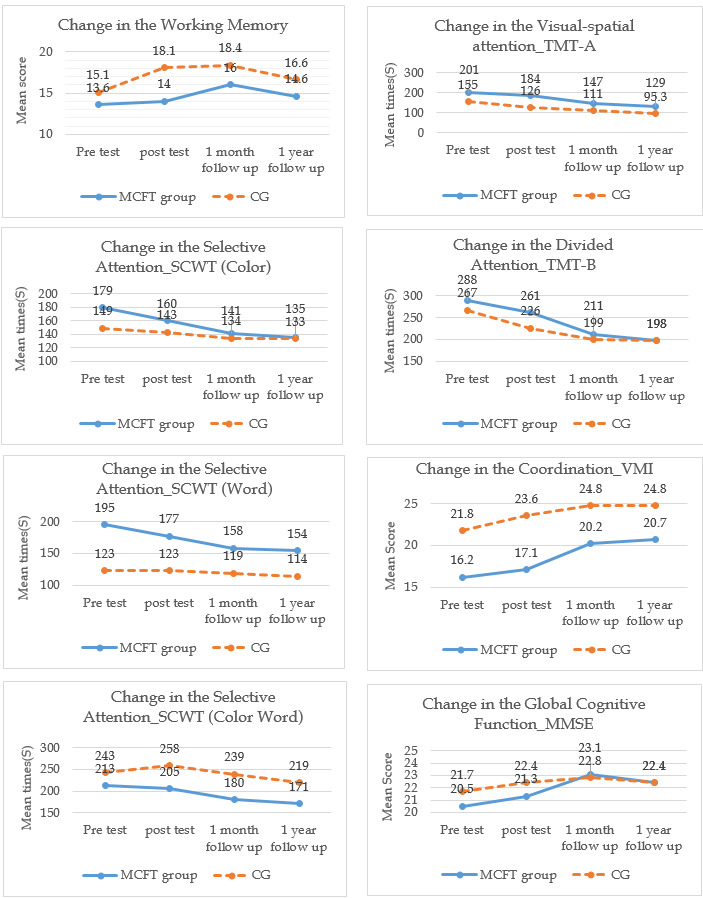
Change score of the outcomes in pretest, post-test, one-month and one-year follow-up.

MMSE scores increased in both groups at both T1 and T2 (**Table 2**, **Figure 2**). GEE showed that the effects of MCFT on global cognitive function remained until one-month follow-up (β =  1.47; 95% CI = 0.63, 2.31; *P* = 0.001) and one-year follow-up (β = 1.51; 95% CI = 0.40, 2.63; *P* = 0.008) after the intervention, with a significant difference (**Table 3**).

**Table 2:** Working memory, attention, coordination and global cognitive function in pretest (T0), immediate post-test (T1), one-month (T2) and one-year follow-up (T3)*

Variables	T0			T1		T2		T3	
	**MCFT**	**CG**	***P*-value**	**MCFT**	**CG**	**MCFT**	**CG**	**MCFT**	**CG**
**Working memory**									
Digit span	13.6 (1.14)	15.1 (0.62)	0.001	14.0 (1.16)	18.1 (1.03)	16.0 (1.12)	18.4 (1.02)	14.6 (1.07)	16.6 (1.07)
DS-forward	9.97 (0.71)	11.6 (0.47)	<0.001	10.2 (0.73)	12.8 (0.52)	11.3 (0.67)	13.2 (0.46)	11.4 (0.68)	11.5 (0.65)
DS-backward	3.58 (0.57)	3.58 (0.37)	0.022	3.86 (0.57)	5.31 (0.70)	4.72 (0.62)	6.17 (0.72)	3.18 (0.57)	5.12 (0.61)
**Selective attention**									
SCWT(C)	179 (14.9)	149 (13.2)	0.237	160 (14.0)	143 (12.6)	141 (10.2)	134 (12.7)	135 (10.0)	133 (10.4)
SCWT (W)	195 (53.9)	123 (12.3)	0.053	177 (47.4)	123 (11.2)	158 (43.6)	119 (11.4)	154 (42.5)	114 (7.68)
SCWT (CW)	213 (20.4)	243 (27.9)	0.002	205 (19.8)	258 (26.4)	180 (19.2)	239 (24.9)	171 (18.2)	219 (21.6)
**Visual-spatial attention**									
TMT-A	201 (15.4)	155 (15.8)	<0.001	184 (15.0)	126 (11.5)	147 (13.1)	111 (8.59)	129 (11.3)	98.3 (7.41)
**Divided attention**									
TMT-B	288 (23.9)	267 (22.6)	0.129	261 (23.8)	226 (20.8)	211 (19.5)	199 (19.4)	198 (18.4)	198 (16.6)
**Coordination**									
VMI	16.2 (0.73)	21.8 (0.75)	<0.001	17.1 (0.73)	23.6 (0.56)	20.2 (0.77)	24.8 (0.43)	20.7 (0.76)	24.8 (0.45)
**Global cognitive function**									
MMSE	20.5 (0.48)	21.7 (0.55)	0.216	21.3 (0.44)	22.4 (0.59)	23.1 (0.50)	22.8 (0.63)	22.4 (0.56)	22.4 (0.56)

MCFT – multi-domain cognitive function training, DS – digit span, SCWT – Stroop colour and word test, TMT – trail making test, VMI – visual-motor integration, MMSE – mini mental state examination

*Data are presented as mean (SD).

**Table 3:** General estimating equation (GEE) analysis of differences between pretest and post-test global cognitive function (n = 72)*

Variable	β	95% CI	SE	Wald’s χ^2^	*P*-value
**Global cognitive function – MMSE**					
Intercept	20.4	18.5, 22.3	0.96	449.8	<0.001
Group (exp)†	-0.61	-2.35, 1.14	0.89	0.46	0.496
Time (second)‡	0.67	0.21, 1.12	0.23	8.23	0.004
Time (third)‡	1.11	0.50, 1.73	0.31	12.5	<0.001
Time (fourth)‡	0.73	0.13, 1.34	0.31	5.64	0.018
Group (exp) x time (second)§	0.14	-0.51, 0.79	0.33	0.17	0.677
Group (exp) x time (third)§	1.47	0.63, 2.31	0.43	11.8	0.001
Group (exp) x time (fourth)§	1.51	0.40, 2.63	0.57	7.05	0.008

MMSE – mini-mental state examination, exp – experimental, GEE – generalised estimating equation, SE – standard error, CI – confidence interval

*Covariate variable: education; “second” – the measurement at immediate post-test, “third” – the measurement at one-month follow-up, “fourth” – the measurement at one-year follow-up.

†Reference is control group.

‡Reference is time (first).

§Reference is group (CON) × time (baseline).

### Effects of intervention on working memory (DST)

DST total scores increased in both groups at T1 and T2 (**Table 2**, **Figure 2**). The working memory domain showed significantly greater improvement for the intervention compared with the control group from T0 to T1, as the DST total score decreased by -2.47 scores on average (95% CI = -3.91, -1.04; *P* = 0.001). The DS-forward decreased by -1.03 scores on average (95% CI = -1.73, -0.33; *P* = 0.004) while the DS- backward decreased by -1.44 scores on average (95% CI = -2.41, -0.48; *P* = 0.003). We observed significant effects of DS-backward at one-month follow-up (β = -1.45; 95% CI = -2.62, -0.27; *P* = 0.016) and one-year follow-up (β = -1.93; 95% CI = -3.33, -0.54; *P* = 0.007) (**Table 4**).

**Table 4:** GEE analysis of differences between pretest and post-test working memory (n = 72)*

Variable	β	95% CI	SE	Wald’s χ^2^	*P*-value
**Working memory**					
DS					
*Intercept*	12.2	9.40, 15.0	1.43	72.8	<0.001
*Group (exp)†*	0.07	-2.75, 2.89	1.44	0.00	0.961
*Time (second)‡*	2.94	1.55, 4.34	0.71	17.1	<0.001
*Time (third)‡*	3.25	1.56, 4.94	0.86	14.3	<0.001
*Time (fourth)‡*	1.44	-0.14, 3.02	0.81	3.20	0.074
Interactions					
*Group (exp) × time (second)*§	-2.47	-3.91, -1.04	0.73	11.4	0.001
*Group (exp) × time (third)*§	-0.81	-2.65, 1.03	0.94	0.74	0.391
*Group (exp) × time (fourth)*§	-0.39	-3.03, 2.26	1.35	0.08	0.776
DS-forward					
*Intercept*	9.95	8.20, 11.7	0.89	124.9	<0.001
*Group (Exp)†*	-0.71	-2.55, 1.13	0.94	0.57	0.450
*Time (second)‡*	1.22	0.59, 1.86	0.32	14.2	<0.001
*Time (third)‡*	1.67	0.90, 2.43	0.39	18.4	<0.001
*Time (fourth)‡*	-0.07	-1.39, 1.26	0.67	0.01	0.921
Interactions					
*Group (exp) × time (second)*§	-1.03	-1.73, -0.33	0.36	8.36	0.004
*Group (exp) × time (third)*§	-0.36	-1.31, 0.59	0.49	0.55	0.457
*Group (exp) × time (fourth)*§	1.50	-0.53, 3.53	1.04	2.10	0.148
DS-backward					
*Intercept*	2.20	0.63, 3.79	0.81	7.49	0.006
*Group (Exp)†*	0.82	-0.70, 2.35	0.78	1.12	0.290
*Time (second)‡*	1.72	0.78, 2.66	0.48	12.9	<0.001
*Time (third)‡*	2.58	1.50, 3.67	0.55	21.8	<0.001
*Time (fourth)‡*	1.55	0.66, 2.44	0.45	11.7	0.001
Interactions					
*Group (exp) × time (second)*§	-1.44	-2.41, -0.48	0.49	8.60	0.003
*Group (exp) × time (third)*§	-1.45	-2.62, -0.27	0.60	5.80	0.016
*Group (exp) × time (fourth)*§	-1.93	-3.33, -0.54	0.71	7.38	0.007

Exp – experimental, DS – digit span, GEE – generalised estimating equation, CI – confidence interval, SE – standard error

*Covariate variable: education; “second” – the measurement at immediate post-test, “third” – the measurement at one-month follow-up, “fourth” – the measurement at one-year follow-up.

†Reference is control group.

‡Reference is time (first).

§Reference is group (CON) × time (baseline).

### Effects of intervention on Selective Attention (SCWT)

SCWT decreased in both groups at T2 (**Table 2**, **Figure 2**). The GEE analysis showed significantly greater improvement in selective attention for the intervention compared with the control group at T2, with the SCWT colour decreasing by -23.3 seconds on average (95% CI = -43.9, -2.76; *P* = 0.026), the SCWT word by -34.2 seconds on average (95% CI = -58.6, -9.82; *P* = 0.006), and the SCWT colour word by -30.2 seconds on average (95% CI -58.5, -1.95; *P* = 0.036). The effects of SCWT colour remained until one-year follow-up (β = -27.8; 95% CI = -47.1, -8.48; *P* = 0.005) with a significant difference (**Table 5**).

**Table 5:** GEE analysis of differences between pretest and post-test selective attention (n = 72)*

Variable	β	95% CI	SE	Wald’s χ^2^	*P*-value
**Selective attention**					
SCWT colour					
*Intercept*	166.6	129.3, 203.8	19.0	76.6	<0.001
*Group (exp)†*	24.8	-15.0, 64.6	20.3	1.50	0.222
*Time (second)‡*	-6.14	-14.4, 2.10	4.20	2.13	0.144
*Time (third)‡*	-14.7	-24.3, -5.08	4.90	8.99	0.003
*Time (fourth)‡*	-16.2	-29.8, -2.70	6.91	5.52	0.019
*Group (exp) × time (second)*§	-12.3	-24.3, -0.30	6.11	4.03	0.045
*Group (exp) × time (third)*§	-23.3	-43.9, -2.76	10.5	4.94	0.026
*Group (exp) × time (fourth)*§	-27.8	-47.1, -8.48	9.86	7.95	0.005
SCWT word					
*Intercept*	154.6	61.9, 247.4	47.3	10.7	0.001
*Group (Exp)†*	89.5	-58.1, 237.1	75.3	1.41	0.234
*Time (second)‡*	0.73	-4.70, 6.16	2.77	0.07	0.793
*Time (third)‡*	-3.56	-9.15, 2.04	2.86	1.55	0.213
*Time (fourth)‡*	-8.75	-19.9, 2.35	5.66	2.39	0.122
*Group (exp) × time (second)*§	-18.3	-33.8, -2.86	7.90	5.39	0.020
*Group (exp) × time (third)*§	-34.2	-58.6, -9.82	12.4	7.56	0.006
*Group (exp) × time (fourth)*§	-31.9	-64.4, 0.56	16.6	3.71	0.054
SCWT color word					
*Intercept*	214.9	155.0, 274.7	30.5	49.5	<0.001
*Group (Exp)†*	-7.47	-76.0, 61.1	34.9	0.05	0.831
*Time (second)‡*	15.6	-12.8, 43.9	14.5	1.16	0.282
*Time (third)‡*	-3.78	-28.1, 20.6	12.4	0.09	0.761
*Time (fourth)‡*	-22.8	-50.3, 4.59	14.0	2.66	0.103
*Group (exp) × time (second)*§	-24.7	-53.4, 3.98	14.6	2.85	0.091
*Group (exp) × time (third)*§	-30.2	-58.5, -1.95	14.4	4.39	0.036
*Group (exp) × time (fourth)*§	-20.0	-50.4, 10.4	15.5	1.66	0.198

SCWT – Stroop Color and Word test, exp – experimental, GEE – generalized estimating equation, SE – standard error, CI – confidence interval

*Covariate variable: education; “second” – the measurement at immediate post-test, “third” – the measurement at one-month follow-up, “fourth” – the measurement at one-year follow-up.

†Reference is control group.

‡Reference is time (first).

§Reference is group (CON) × time (baseline).

### Effects of intervention on visual-spatial and divided attention (TMT A and B)

The GEE analysis showed that visual-spatial and divided attention outcomes of the experimental group did not improve post-training (**Table 6**).

**Table 6:** GEE analysis of differences between pretest and post-test visual-spatial attention, and divided attention (n = 72)

Variable	β	95% CI	SE	Wald’s χ^2^	*P*-value
**Visual-spatial attention**					
TMT-A					
*Intercept*	213.4	167.7, 259.0	23.3	84.0	0.001
*Group (exp)†*	15.8	-28.0, 59.6	22.4	0.50	0.479
*Time (second)‡*	-28.3	-42.6, -14.0	7.29	15.1	<0.001
*Time (third)‡*	-43.7	-66.3, -21.0	11.5	14.3	<0.001
*Time (fourth)‡*	-56.3	-80.8, -31.9	12.5	20.4	<0.001
*Group (exp) × time (second)*§	11.9	-3.26, 27.1	7.76	2.37	0.124
*Group (exp) × time (third)*§	-10.2	-36.6, 16.2	13.5	0.57	0.450
*Group (exp) × time (fourth)*§	-16.5	-45.7, 12.8	14.9	1.22	0.270
**Divided attention**					
TMT-B					
*Intercept*	307.8	200.1, 415.4	54.9	31.4	<0.001
*Group (Exp)†*	1.43	-85.6, 88.4	44.4	0.001	0.974
*Time (second)‡*	-41.3	-54.5, -28.1	6.72	37.8	<0.001
*Time (third)‡*	-68.4	-86.3, -50.4	9.16	55.7	<0.001
*Time (fourth)‡*	-68.5	-96.6, -40.3	14.4	22.7	<0.001
*Group (exp) × time (second)*§	14.1	-5.40, 33.6	9.95	2.01	0.156
*Group (exp) × time (third)*§	-9.45	-49.0, 30.1	20.2	0.22	0.640
*Group (exp) × time (fourth)*§	-23.4	-75.6, 28.8	26.6	0.77	0.380

TMT – trail making test, exp – experimental, GEE – generalized estimating equation, SE – standard error, CI – confidence interval

*Covariate variable: education; “second” – the measurement at immediate post-test, “third” – the measurement at one-month follow-up, “fourth” – the measurement at one-year follow-up.

†Reference is control group.

‡Reference is time (first).

§Reference is group (CON) × time (baseline).

### Effects of intervention on coordination (VMI)

VMI scores increased in both groups at T0 and T3 (**Table 2**, **Figure 2**). From T0 to T1, the VMI of the experimental group decreased by -0.89 scores on average (*P* = 0.045) compared with the control group. The effects of VMI remained until one-year follow-up (β = 1.61; 95% CI = 0.25-2.96; *P* = 0.020) with the difference being significant (**Table 7**).

**Table 7:** GEE analysis of differences between pretest and post-test coordination (n = 72)

Variable	β	95% CI	SE	Wald’s χ^2^	*P-*value
**Coordination**					
VMI					
*Intercept*	19.6	17.3, 21.8	1.16	285.4	<0.001
*Group (Exp)†*	-4.24	-6.35, -2.13	1.07	15.6	0.211
*Time (second)‡*	1.83	1.11, 2.56	0.37	24.3	<0.001
*Time (third)‡*	3.00	2.11, 3.90	0.46	43.2	<0.001
*Time (fourth)‡*	2.97	2.05, 3.89	0.47	40.1	<0.001
Interactions					
*Group (exp) × time (second)*§	-0.89	-1.76, -0.02	0.44	4.02	0.045
*Group (exp) × time (third)*§	1.06	-0.30, 2.42	0.69	2.32	0.128
*Group (exp) × time (fourth)*§	1.61	0.25, 2.96	0.69	5.38	0.020

Exp – experimental, VMI – visual-motor integration, SE – standard error, CI – confidence interval

*Covariate variable: education; “second” – the measurement at immediate post-test, “third” – the measurement at one-month follow-up, “fourth” – the measurement at one-year follow-up.

†Reference group: control group.

‡Reference group: time (first).

§Reference group (CON) x time (baseline).

## DISCUSSION

We examined the efficacy of multi-domain cognitive function training for 30 minutes at three times a week for eight weeks and found it to be effective in improving cognitive function, working memory, attention, and coordination of older adults with mild cognitive impairment and mild dementia. No side effects were reported during training. The effects of multi-domain cognitive function training remained for one year after training. However, we found no significant improvement for visual-spatial and divided attention. This aligns with the STAC, which is based on the principles of neuroplasticity and cognitive reserve. When cognitive decline occurs, new neural connections can be generated to compensate for old and weakened neurons and thereby maintain basic cognitive function. Thus, proactive behaviours that improve brain performance including cognitive training by helping older adults develop an efficient scaffolding model, increase brainpower and cognitive reserve, and delay cognitive aging are needed [18]. As there currently is no set standard for clinical significance, we urge clinicians to consider the reported effect size and confidence intervals in our results sections, but we have compared our results with previous research in the following sections.

### Participant demographics

We have found significant differences in education and borderline differences in sex. The GEE model adjusted for education and found that it did not have an impact on the study results. Sex differences in dementia are also associated with brain reserves. Brain reserves refer to the brain's ability to maintain sufficient functioning in the face of ongoing damage. Literature reviews also seem to support the hypothesis that females have lower brain reserves and are more likely to manifest Alzheimer disease pathological symptoms clinically, while males are more resilient to its pathological process [34-36]. However, females are more susceptible to cognitive impairment, which also suggests that they are more vulnerable to the impact of cognitive impairments in the early stages. We have yet to fully understand the biological mechanisms behind these differences, but they partly reflect the following: traditionally, males have had more opportunities for formal education, good occupations, and leisure activities, which may contribute to their greater brain reserves. Generally, older males tend to be more active than older female, particularly in physical activities. Engaging in leisure activities can help prevent the onset of dementia, especially those involving physical, mental, and social aspects [37]. Clinicians might consider being more attentive of female older adults with mild cognitive impairment and mild dementia who may need more cognitive training to maintain cognitive function. However, as our result is borderline, clinical discretion is needed.

### Effect of MCFT on global cognitive function

Our results indicate that multi-domain cognitive function training had a moderate significant effect on global cognitive function, and are thus in line with a meta-analysis [38] which included older adults with mild cognitive impairment from 17 randomised controlled trials. Another study [39] demonstrated that eight to 12 weeks of computerised cognitive training improved immediate memory; this might be due to the study examining older adults with mild cognitive impairment and mild dementia. We observed satisfactory results after eight weeks of 30-minute training sessions three times a week, which might be relevant for clinicians.

### Effect of MCFT on working memory

Working memory can be used to assess an individual’s ability to process information. We used the DST to test the participants’ maximum memory capacity for a specific type of information in a short period. DS-forward is a representative test of sequential information processing, which is often used in daily life. DS-backward involves higher-level information processing and information storage. Because these mechanisms are not commonly used, the interventions’ effects were high and clinically relevant. The results are in line with those of other studies [13,40-42] in which cognitive training had a moderate to large effect size on working memory, indicating that interventions could improve and maintain working memory. Clinicians should recognise that declines in memory and working memory are the earliest and most pronounced type of cognitive decline because working memory is a higher-order cognitive ability and more susceptible to the effects of aging [3]. Notably, the use of cognitive training interventions can activate the frontal lobe improving attention and memory, thereby promoting brain neuroplasticity [4].

### Effect of MCFT on attention

Physiological changes caused by aging increase the time older adults require to process information and react to their environment. Low attention levels in patients with mild cognitive impairment can lead to a loss of the ability to perform activities of daily living. Thus, cognitive training may help mitigate the negative effects of aging on attention. Game-based intelligence tests have demonstrated to increase neuroplasticity and older adults’ interest in learning. The participants in the multi-domain cognitive function training group exhibited improvements in selective attention but no significant improvement was observed for visual-spatial and divided attention. Clinicians should be aware that these results are inconsistent with those of Yang et al. [10] who conducted training on selective, focused, and divided attention, and those of Yang et al. [11] who conducted multi-domain attention training on alertness, sustained attention, and visual-spatial attention. This difference might be due to these studies conducting computerised cognitive training and using attention as the primary training domain, while we adopted a game-based intelligence test to measure visual-spatial (TMT-A) and divided attention (TMT-B). Both the training and test required the participants to use their visual search abilities to identify numbers and the 12 zodiac signs, which is why the two tests did not reveal significant effects. For the participants, age and visual load are crucial factors that affect visual-spatial attention. Studies have found that visual-spatial attention is associated with motor skills and older adults with a history of falls may develop visual-spatial attention deficits [40,41]. Improving visual-spatial attention can reduce older adults’ risk of falls, prevent them from becoming disoriented in familiar environments, and decrease forgetfulness. Because attention training requires prolonged focus on a specific task, age and visual load should be considered to shorten training time, and the number of training sessions should be increased.

### Effect of MCFT on coordination

Coordination is key to performing independent functions well. A decline in VMI is often observed in the early stages of cognitive decline [43]. For complete VMI, several brain regions must be activated to recognise visual stimuli and execute movements. The occipital, parietal, and temporal lobes differentiate and identify visual stimuli. The frontal lobe processes sensory information and responds with the appropriate behaviour, and the basal ganglia and cerebellum control muscle movement. VMI training can stimulate all regions of the brain and increase neuroplasticity, thereby facilitating performance of activities of daily living. We improved participants’ coordination by training their VMI through dynamic target tracking and synchronised hand-tapping movements, with similar results to Chan et al. [7]. Physical fitness, agility, and proprioception decrease with age, resulting in poor coordination. Studies have demonstrated that VMI training is an effective method of enhancing the cognitive function of older adults and that VMI interventions are crucial to preventing cognitive decline [44]. This is clinically relevant, as such training also strengthens older adults’ abilities to pick up and manipulate everyday objects and to control movement.

### Strengths and limitations

Our trial had numerous strengths, the first being the use of multi-domain cognitive function training instead of a single domain, which reported improvements in cognition. Second, we had a rigorous research design, with clear inclusion and exclusion conditions and the use of research tools with good reliability and validity to measure the research results and obtain the initial effect of intervention measures. Third, we applied the intention-to-treat principle, which includes all randomly assigned participants, rather than just those who completed the trial. However, our study had limitations. We drew the participants from remote areas in the northern region with limited healthcare resources. Due to the COVID-19 pandemic, we had a small sample size, and social distancing measures may have prevented older adults from participating in certain group activities. Additionally, concerns about the risk of infection among family members and the health issues faced by older adults, such as chronic diseases and declining physical function, may have resulted in a reduction of approximately 20% in the sample size. Future studies should consider using larger sample sizes and conducting longitudinal long-term follow-up to evaluate the longer-term effects of multi-domain cognitive training. We focused on older adults with mild cognitive impairment and mild dementia, so subsequent studies are needed across populations and locations in various regions to improve and increase the generalisability of our findings.

## CONCLUSIONS

Our findings demonstrate that multi-domain cognitive function training produce favourable effects, improving global cognitive function, working memory, selective attention, and coordination among older adults with mild cognitive impairment and mild dementia. Therefore, incorporating this training for such populations in community care centres can be a strategy for delaying cognitive decline. We suggest that nurses working in community healthcare settings conduct regular activities and long-term training to delay cognitive decline among older adults.
